# Performance of VIDISCA-454 in Feces-Suspensions and Serum 

**DOI:** 10.3390/v4081328

**Published:** 2012-08-22

**Authors:** Michel de Vries, Bas B. Oude Munnink, Martin Deijs, Marta Canuti, Sylvie M. Koekkoek, Richard Molenkamp, Margreet Bakker, Suzanne Jurriaans, Barbera D. C. van Schaik, Angela C. Luyf, Silvia D. Olabarriaga, Antoine H. C. van Kampen, Lia van der Hoek

**Affiliations:** 1 Laboratory of Experimental Virology, Department of Medical Microbiology, Center for Infection and Immunity Amsterdam (CINIMA), Academic Medical Center of the University of Amsterdam, 1105AZ, Amsterdam, The Netherlands; Email: micheldevr@gmail.com (M.V.); b.b.oudemunnink@amc.uva.nl (B.B.O.M.); m.deijs@amc.uva.nl (M.D.); m.canuti@amc.uva.nl (M.C.); m.e.bakker@amc.uva.nl (M.B.); 2 Laboratory of Clinical Virology, Department of Medical Microbiology, Center for Infection and Immunity Amsterdam (CINIMA), Academic Medical Center of the University of Amsterdam, 1105AZ, Amsterdam, The Netherlands; Email: s.m.koekkoek@amc.uva.nl (S.M.K.); r.molenkamp@amc.uva.nl (R.M.); s.jurriaans@amc.uva.nl (S.J.); 3 Department of Clinical Epidemiology, Biostatistics and Bioinformatics, Bioinformatics Laboratory, Academic Medical Center of the University of Amsterdam, 1105AZ, Amsterdam, The Netherlands; b.d.vanschaik@amc.uva.nl (B.D.C.S.); a.c.luyf@amc.uva.nl (A.C.L.); S.D.Olabarriaga@amc.uva.nl (S.D.O.); a.h.vankampen@amc.uva.nl (A.H.C.K.); 4 Biosystems Data Analysis, Swammerdam Institute for Life Sciences, University of Amsterdam, Science Park 904, 1098 XH, Amsterdam, The Netherlands

**Keywords:** virus discovery, VIDISCA, diarrhoea, HIV-1, norovirus

## Abstract

Virus discovery combining sequence unbiased amplification with next generation sequencing is now state-of-the-art. We have previously determined that the performance of the unbiased amplification technique which is operational at our institute, VIDISCA-454, is efficient when respiratory samples are used as input. The performance of the assay is, however, not known for other clinical materials like blood or stool samples. Here, we investigated the sensitivity of VIDISCA-454 with feces-suspensions and serum samples that are positive and that have been quantified for norovirus and human immunodeficiency virus type 1, respectively. The performance of VIDISCA-454 in serum samples was equal to its performance in respiratory material, with an estimated lower threshold of 1,000 viral genome copies. The estimated threshold in feces-suspension is around 200,000 viral genome copies. The decreased sensitivity in feces suspension is mainly due to sequences that share no recognizable identity with known sequences. Most likely these sequences originate from bacteria and phages which are not completely sequenced.

## 1. Introduction

The discovery of viral pathogens has been intensified in the last decades by improved molecular methods (SISPA [1], random priming based assays [2], universal primers [3] and representational difference analysis [4]). With the introduction of high throughput sequencing, the chance and possibilities of identifying a new virus increased even more. The costs per nucleotide sequenced reduced dramatically, whereas the amount of data retrieved from a single experiment increased massively. Consequently, retrieving thousands of sequence reads from a single sample allows, in theory, identification of a virus even with moderate or low viral loads. The unbiased virus discovery amplification method used at our institute is VIDISCA, a restriction enzyme recognition based amplification technique that allows amplification of RNA or DNA sequences regardless of the genome composition [5]. The PCR products of VIDISCA can easily be sequenced via next generation sequencing techniques when adaptors carrying the Roche-454 necessary tail (A and B tails) are ligated during VIDISCA. The VIDISCA-454 assay allows virus amplification in respiratory clinical samples without the necessity to culture [6,7]. No more than +/− 5,000 reads from a respiratory sample are needed to have a satisfactory assay sensitivity of VIDISCA-454 [6]. However, the question is if the method is sensitive enough for other clinical materials. A virus hunt to identify novel viruses involved in various diseases, for example undiagnosed diarrhea or Kawasaki disease, will have to be performed in materials other than respiratory samples. Most likely, these clinical materials have different sample specific background, e.g., feces will contain a lot of bacteria that might lead to a reduced amplification of viral sequences. Therefore, it is necessary to determine whether viral fragments can be detected in non-respiratory clinical samples. In this study we focused on the performance of VIDISCA-454 in fecal material and serum samples.

## 2. Results and Discussion

**Figure 1:**
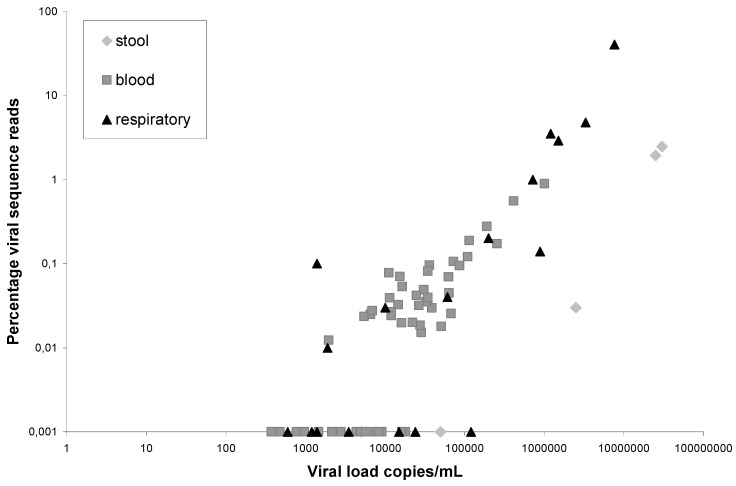
Performance of VIDISCA-454 in feces suspensions (◊), serum (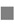), and respiratory copan-collected swabs (▲). On the X-axis the viral load in a clinical sample is indicated, on the Y-axis the percentage of viral sequence reads: HIV-1 (blood), norovirus (stool), coronaviruses, adenoviruses, picornaviruses and influenzaviruses (respiratory material). The viruses and percentages in the respiratory samples have been published previously [6].

The performance of VIDISCA-454 in feces was investigated using broth suspensions from a sample-bank collected from HIV-1 infected individuals in 1994 and 1995 [8]. A norovirus infection was diagnosed in five people via real time RT-PCR [9], the viral load in feces suspension ranged between 5 × 10^3^ and 3 × 10^7^ genome copies/mL. VIDISCA-454 was performed as described previously for respiratory material [6], thus no extra purification methods like filtration were performed. In total, 22,661 sequences were obtained for these five patients and 225 of them were noroviral sequences. These sequences were present in the reads from three out of five positive patients. The three VIDISCA-454 positive samples had a viral load of 2.5 × 10^7^, 3.0 × 10^7^ and 2.5 × 10^6^ genome copies/mL, and 103 out of 5,301, 121 out of 4,909, and 1 out of 3,317 sequences were from noroviruses respectively (percentages are shown in Figure 1). The two samples which were negative had lower copy numbers, 5 × 10^4^ and 5 × 10^3^ genome copies/mL, and no norovirus sequence was detectable in the 4,160 and 4,974 reads respectively. Subsequently, the background sequences were examined to determine the source of the background. Surprisingly, hardly any known sequence acts as main competitor: about 80% of the reads are “no-hits”. No-hits are sequences with good quality and an average length that share no recognizable homology with known sequences of the non-redundant NCBI database. The remaining 20% mainly consisted of bacterial sequences, probably originating from the gut microbiota.

The performance of VIDISCA in blood products was investigated with sera from human immunodeficiency virus type 1 (HIV-1) infected persons (n = 54). The viral load varied between 1.5 × 10^2^ and 1 × 10^6^, with a mean of 5.6 × 10^4^ RNA copies/mL. In total 278,944 VIDISCA-454-reads were retrieved, and 178 of them were HIV sequences. The 178 sequences originated from 36 out of 54 samples (65%). The samples in which HIV-1 was not detected by VIDISCA-454 had low HIV-1 copy numbers (range 2 × 10^3^ and 1.8 × 10^4^; mean 5.4 × 10^3^ RNA copies/mL), whereas the VIDISCA-454 positive samples ranged in viral load between 2 × 10^3^ and 1 × 10^6^ (mean 8.3 × 10^4^). The plot with the percentage of viral hits in comparison to the viral load is shown in Figure 1. It illustrates a trend: an increased percentage of viral sequences with higher viral loads. For comparison, the relationship between viral load and percentage viral sequences for respiratory material and fecal material is also included in Figure 1, and we observe that, in general, serum samples and respiratory samples have the same performance. For respiratory material, the background in VIDISCA-454 mainly consists of human ribosomal RNA (rRNA). In serum, the background is primarily of eukaryotic origin (33%), probably originating from human DNA, and only 9.5% of all sequences were from rRNA. Furthermore, a substantial amount of sequences were detected that originate from VIDISCA ingredients (vectors that produce the enzymes, *etc.*). This shows that the material is relatively clean and requires no further purification. We can exclude the possibility that the vectors were introduced in the clinical sample in our laboratory since most of the encountered vectors were never used.

## 3. Experimental Section

### 3.1. Clinical Samples

Fecal samples were selected from a sample bank containing 56 HIV-1-infected adult patients with diarrhea, aged above 17 who visited the out-patients clinic at the Academic Medical Center in the years 1994–1995 [10]. Fecal samples were suspended in broth (1:3 dilution). Serum samples of HIV-1 infected individuals were obtained from the Amsterdam Cohort Studies on HIV infection and AIDS. Serum sample selection was based on being HIV-1 positive at entry of the Amsterdam Cohort Studies, CD4 counts below 300 cells/mm^3^, and at least 2 years since entry of the Amsterdam Cohort Studies. Needle sharing was the most likely risk factor for HIV-1 infection. The Amsterdam Cohort Studies has been conducted in accordance with the ethical principles set out in the declaration of Helsinki and written informed consent has been obtained prior to data collection. The study was approved by the Amsterdam Medical Center institutional medical ethics committee. 

### 3.2. Real Time HIV-1-RNA and Norovirus-RNA RT-PCR

Viral load of HIV-1 in serum was determined via real time RT-PCR (Abbott RealTime HIV-1 assay; Abbott Molecular), and the fecal samples positive for norovirus were identified by real time RT-PCR using primers Noro-G1-F ATG TTC CGC TGG ATG CG, Noro-G1-R CGT CCT TAG ACG CCA TCA TC, Noro-G2-F CAA GAI CCI ATG TTY AGI TGG ATG AG, Noro-G2-R TCG ACG CCA TCT TCA TTC AC, and Minor Groove Binding probes Noro-G1-P TGG ACA GGA GAT CGC and Noro-G2-P TGG GAG GGC GAT CG with assay conditions as described previously [9].

### 3.3. VIDISCA-454

Cell debris, bacteria and mitochondria were removed from 110 µL feces suspension or serum by centrifugation at 10,000 × g for 10 minutes. Residual DNA was degraded with 20 U TURBO^TM^ DNase (Ambion). Virion-protected nucleic acids were extracted by Boom isolation [11], and elution of nucleic acids was performed in sterile H_2_O containing 10 µM of rRNA-blocking oligonucleotides (4 µM each, see [6]). The reverse transcription and VIDISCA-454 were performed as described previously [6] with the following conditions: Reverse transcription was performed with Superscript II (200 U, Invitrogen) in a mixture containing *E. coli* ligase (5 U, Invitrogen) and non-rRNA binding hexamers [12]. The anchor ligation was performed with anchors based on primer A with an identifier sequence (MIDs of 10 nt, see GS FLX Shotgun DNA Library Preparation Method Manual) and 1 anchor containing primer B. In total 14 different identifier sequences were used, allowing 14 samples to be pooled on one region of a picotiterplate. Amplification by PCR, purification of products and emulsion PCR are performed as described [6]. Samples were run on a 4 regions picotiterplate for the 454 Titanium system and processed according to the emulsion small volume PCR protocol with 2 E6 beads per emulsion as input and 4 small volume emulsions per region (direct titration protocol). Reads were assembled using the Codon Code software (version 3.6.1, Condoncode Corporation Dedham, MA, USA). The search for viral sequences was performed with BLAST [13] on the Dutch e-Science Grid with the e-BioInfra platform [14]. This BLAST implementation can take sequences in SFF and FASTA format and performs a search against the GenBank non-redundant nucleotide database [15] in parallel for all input data. For this study we used GenBank build 12-03-2012 and the following parameter settings: -gapopen 5 -gapextend 2 -reward 2 -evalue 1 × 10^−5^.

## 4. Conclusions

Every clinical sample has its own background of VIDISCA-interfering non-viral nucleic acid. For respiratory swabs, we determined that the background mainly consists of rRNA, and we have introduced several adjustments to the VIDISCA protocol to select and enhance sequencing of everything that is different from rRNA [6]. The background of other kinds of materials is unknown therefore we determined the performance and the background in feces suspensions and serum. In general, the VIDISCA-454-performance in serum and respiratory material is comparable with the same detection limit of approximately 10,000 copies/mL. Remarkably, the background in serum is quite different from respiratory material. In respiratory material, the amount of ribosomal RNA is 35%, while it is only 9.5% in serum samples. Careful inspection of the non-viral sequences showed that a substantial amount of the sequences is unassigned, so without any homology with known sequences. In serum, the percentage no-hits is about 10% and in fecal material 80%. This high amount of no-hits in feces could be attributed to the high number of bacteria and phages in this material, many of which have not been fully sequenced and thus the reads remain unassigned.

## References

[1] Allander T., Emerson S.U., Engle R.E., Purcell R.H., Bukh J. (2001). A virus discovery method incorporating DNase treatment and its application to the identification of two bovine parvovirus species. Proc. Natl. Acad. Sci. USA.

[2] Kapoor A., Slikas E., Simmonds P., Chieochansin T., Naeem A., Shaukat S., Alam M.M., Sharif S., Angez M., Zaidi S. (2009). A newly identified bocavirus species in human stool. J. Infect. Dis..

[3] Woo P.C., Lau S.K., Huang Y., Tsoi H.W., Chan K.H., Yuen K.Y. (2005). Phylogenetic and recombination analysis of coronavirus HKU1, a novel coronavirus from patients with pneumonia. Arch. Virol..

[4] Chang Y., Cesarman E., Pessin M.S., Lee F., Culpepper J., Knowles D.M., Moore P.S. (1994). Identification of herpesvirus-like DNA sequences in AIDS-associated Kaposi’s sarcoma. Science.

[5] Van der Hoek L., Pyrc K., Jebbink M.F., Vermeulen-Oost W., Berkhout R.J., Wolthers K.C., Wertheim-van Dillen P.M., Kaandorp J., Spaargaren J., Berkhout B. (2004). Identification of a new human coronavirus. Nat. Med..

[6] De Vries M., Deijs M., Canuti M., van Schaik B.D., Faria N.R., van de Garde M.D., Jachimowski L.C., Jebbink M.F., Jakobs M., Luyf A.C. (2011). A sensitive assay for virus discovery in respiratory clinical samples. PLoS One.

[7] Canuti M., Eis-Huebinger A.M., Deijs M., de Vries M., Drexler J.F., Oppong S.K., Muller M.A., Klose S.M., Wellinghausen N., Cottontail V.M. (2011). Two novel parvoviruses in frugivorous New and Old World bats. PLoS One.

[8] Van der Hoek L., Sol C.J., Maas J., Lukashov V.V., Kuiken C.L., Goudsmit J. (1998). Genetic differences between human immunodeficiency virus type 1 subpopulations in faeces and serum. J. Gen. Virol..

[9] Jansen R.R., Schinkel J., Koekkoek S., Pajkrt D., Beld M., de Jong M.D., Molenkamp R. (2011). Development and evaluation of a four-tube real time multiplex PCR assay covering fourteen respiratory viruses, and comparison to its corresponding single target counterparts. J. Clin. Virol..

[10] Snijders F., Kuijper E.J., de Wever B., van der Hoek L., Danner S.A., Dankert J. (1997). Prevalence of Campylobacter-associated diarrhea among patients infected with human immunodeficiency virus. Clin. Infect. Dis..

[11] Boom R., Sol C.J., Salimans M.M., Jansen C.L., Wertheim-van Dillen P.M., van der Noordaa J. (1990). Rapid and simple method for purification of nucleic acids. J. Clin. Microbiol..

[12] Endoh D., Mizutani T., Kirisawa R., Maki Y., Saito H., Kon Y., Morikawa S., Hayashi M. (2005). Species-independent detection of RNA virus by representational difference analysis using non-ribosomal hexanucleotides for reverse transcription. Nucleic Acids Res..

[13] Altschul S.F., Gish W., Miller W., Myers E.W., Lipman D.J. (1990). Basic local alignment search tool. J. Mol. Biol..

[14] Luyf A.C., van Schaik B.D., de Vries M., Baas F., van Kampen A.H., Olabarriaga S.D. (2010). Initial steps towards a production platform for DNA sequence analysis on the grid. BMC Bioinformatics.

[15] Benson D.A., Karsch-Mizrachi I., Clark K., Lipman D.J., Ostell J., Sayers E.W. (2012). GenBank. Nucleic Acids Res..

